# Cultural Protection from Polysubstance Use Among Native American Adolescents and Young Adults

**DOI:** 10.1007/s11121-022-01373-5

**Published:** 2022-06-01

**Authors:** Teresa N. Brockie, Jacquelyn C. Campbell, Gail Dana-Sacco, Jason Farley, Harolyn M. E. Belcher, Joan Kub, Katie E. Nelson, Jerreed D. Ivanich, Li Yang, Gwenyth Wallen, Lawrence Wetsit, Holly C. Wilcox

**Affiliations:** 1 Johns Hopkins University School of Nursing, 525 N. Wolfe Street Room N530M, Baltimore, MD USA; 2 Center for American Indian Health, Johns Hopkins Bloomberg School of Public Health, Baltimore, MD USA; 3 Kennedy Krieger Institute, Baltimore, MD USA; 4 Centers for American Indian and Alaska Native Health, Department of Community and Behavioral Health, University of Colorado Anschutz Medical Campus, Aurora, CO USA; 5 Clinical Center, National Institutes of Health, Bethesda, MD USA; 6 Fort Peck Community, Wolf Point, Chicago, MT USA; 7 Department of Mental Health, Johns Hopkins Bloomberg School of Public Health, Baltimore, MD USA

**Keywords:** Polysubstance use, Drug use, Substance use, Cultural protective factors, Native American, Survey methodology

## Abstract

Reservation-based Native American youth are at disproportionate risk for high-risk substance use. The culture-as-treatment hypothesis suggests aspects of tribal culture can support prevention and healing in this context; however, the protective role of communal mastery and tribal identity have yet to be fully explored. The objectives of this study were to investigate (1) the relationship between cultural factors and high-risk substance use, which includes polysubstance use, early initiation of alcohol and illicit drugs, and binge drinking, and (2) substance use frequency and prevalence of various substances via cross-sectional design. Multiple logistic regression modeling was used to analyze data from 288 tribal members (15–24 years of age) residing on/near the Fort Peck Reservation in the Northern Plains. When controlling for childhood trauma and school attendance, having at least a high school education (OR = 0.434, *p* = 0.028), increased communal mastery (OR = 0.931, *p* = 0.007), and higher levels of tribal identity (OR = 0.579, *p* = 0.009) were significantly associated with lower odds of polysubstance use. Overall prevalence of polysubstance use was 50%, and binge drinking had the highest single substance prevalence (66%). Prevalence of early initiation of substances (≤ 14 years) was inhalants (70%), alcohol (61%), marijuana (74%), methamphetamine (23%), and prescription drug misuse (23%). Hydrocodone, an opioid, was the most frequently misused prescription drug. Findings indicate programs focused on promoting education engagement, communal mastery, and tribal identity may mitigate substance use for Native American adolescents living in high-risk, reservation-based settings.

## Introduction

National prevalence estimates for lifetime, recent, and current drug use (such as of methamphetamine, marijuana, prescription drug misuse, inhalants, and problematic alcohol use) are higher for Native American (American Indian/Alaska Native) adolescents and young adults when compared to the general population (Substance Abuse Mental Health Services Administration [SAMHSA], 2019a). Substance use among Native American youth varies significantly by geography (Indian Health Service [IHS], 2012); but overall, reservation-based Native American youth are at significantly higher risk for problematic substance use. They are more likely to use substances at an earlier age, engage in risky drinking behaviors (such as binge drinking), experience alcohol dependence symptoms, and possess higher rates of substance use when compared to their urban Native American counterparts and the general population (Beauvais, 1992; Stanley & Swaim, 2015; Tingey et al., 2016; Walls et al., 2013; Whitesell et al., 2014; Yu & Stiffman, 2007).

There are multiple factors that place Native American adolescents at increased risk for substance use. Factors include poverty, opportunities to use alcohol and drugs (such as availability of substances in school, peer substance use), lack of parental supervision, and early onset aggressive behavior (National Institute on Drug Abuse [NIDA], 2003). Communities with lower educational attainment, high unemployment, and poverty typically have greater prevalence of substance use and earlier initiation of use among youth (Grant et al., 2016; Ramisetty-Mikler & Ebama, 2011). These factors are highly prevalent among reservation communities with rates of poverty around 32% — nearly three times that of the general population, and with per capita income nearly half that of the general population (Akee & Taylor, 2014). Furthermore, five of the ten poorest counties in the United States (U.S.) are home to an Indian reservation (MainStreet, 2011). Evidence supports that substance use during adolescence — a critical period of development — often leads to developmental continuity, whereby individuals continue to use drugs into adulthood and tend to become polysubstance users (Felton et al., 2015).

Further, Native American youth suffer the highest burden of childhood trauma and suicide of any racial group in the U.S. (Willmon-Haque & BigFoot, 2008). Mental health problems such as depression, childhood trauma, substance use, impulsivity, loss of cultural identity, low self-esteem, and hopelessness are key risk factors prevalent in this population (Willmon-Haque & BigFoot, 2008). Health system barriers, including scarcity of mental health services and providers, particularly Indigenous providers, and lack of tribal-specific data for informing development also negatively impact Native trauma and suicide rates (Brockie et al., 2021). However, unique Native American cultural understandings of mental health, culturally informed protective factors, and a preference for culturally based healing modalities are underdeveloped assets, often stymied by Western care systems (Brockie et al., 2021).

Problematic substance use and/or polysubstance use may be exacerbated in some Native American communities due to intergenerational social disadvantage, economic marginalization, legacies of historical injustices, and lack of access to timely and culturally appropriate treatment options (Acevedo et al., 2018; Beals et al., 2003; Broffman et al., 2017). Furthermore, policies enforcing acculturation, such as the mandatory boarding schools, were collectively traumatic and underpinned the loss of tribal identity and cultural beliefs (Beauvais, 1998; Brave Heart, 1996; Brave Heart & DeBruyn, 1998; Duran et al., 1998). Reservation-based youth who attended government-run boarding schools or who have dropped out of school have been identified as having increased use of alcohol (Beauvais, 1998). In reservation settings, comorbid substance abuse and mental health disorders are widespread and unfortunately contribute to a disproportionate rate of incarcerations among these communities, further perpetuating the cycle of poverty (Hartshorn et al., 2015).

High-risk substance use includes polysubstance use, early initiation, and binge drinking. Alcohol, marijuana, and inhalants have been identified as common initiation substances among Native American adolescents (Novins et al., 2011). Early initiation (≤ 14 years of age) of alcohol and illicit drug use is associated with multiple risk behaviors in adolescence and is linked to long-term health risks (SAMHSA, 2014). Early onset substance use is associated with increased conduct disorder, depressive symptoms, and subsequent polysubstance use among adolescents (Kunitz, 2008; Stanley & Swaim, 2015; Strunin et al., 2017; Wymbs et al., 2014); it is also a risk factor for suicide (Turecki & Brent, 2016). Those who begin using alcohol at a younger age are more likely to continue into adulthood and are at risk for alcohol abuse and dependence (May, 1996), rendering them at higher risk for psychiatric disorders (Grant et al., 2016), suicidal ideation, and suicide attempts.

Binge drinking is a pattern of excessive alcohol intake that results in a person’s blood alcohol level reaching 0.08% (National Institute on Alcohol Abuse and Alcoholism [NIAAA], 2020). For men, this threshold is reached after consuming five or more drinks, and four or more drinks for women, in a span of 2 h (NIAAA, 2020). Adolescent binge drinking is frequently linked to alcohol poisoning, fatal accidents and associated injuries, and increased risk of developing alcohol use disorder later in life (Kiedrowski & Selva, 2019; SAMHSA, 2019b; Stanley & Swaim, 2018). Use of multiple substances is also linked with a greater likelihood of overdose (Silva et al., 2013); decreased cognitive functioning (Connor et al., 2014); psychiatric comorbidity (Lynskey et al., 2007); and high-risk sexual behavior (McCarty-Caplan et al., 2013). Among Native American adolescent samples, polysubstance use is higher among Northern Plains tribes than Southwest tribes and is linked to substance dependence (Whitesell et al., 2006), decreased odds of full-time employment, poor physical health, and lower levels of income (Sittner et al., 2021). Polysubstance use is also associated with binge drinking (Conway et al., 2013).

Polysubstance describes a variety of substance use disorders; it is commonly classified concurrently as polydrug use (two or more substances within a given period) or simultaneous drug use (two or more substances at the same time) (Martin, 2008). Although it is relatively common among adolescents, polysubstance use is associated with poor mental health outcomes (Kelly et al., 2015; Martin, 2008), trauma, and suicide risk (DiGuiseppi et al., 2020). Available research uses different data sources and definitions of polydrug use, yet there are inconsistent findings in Native American youth and research on sequencing and combinations of polydrug use is scarce. Schick et al. (2021) and Banks et al (2021) both reported a dearth of research on polydrug use among Native adolescents despite the harmful consequences, including risky sexual behavior, psychological distress, greater likelihood of school disengagement and dropout, and drug use in adulthood. Kiedrowski and Selva (2019) used 2013 national YRBS data showing that distinct patterns of polysubstance use exist between Native American and White youth, with Natives reporting use of more steroids and injected drugs than White youth. Banks and colleagues (2021) used NSDUH data which differed from other studies, as it did not show many differences in polydrug use among Native American youth. Overall, there are few studies examining polydrug use in Native American youth and the results on patterns and sequencing are inconsistent.

There is variability in substance use within Native American populations; however, it has generally been considered an acceptable practice (Kunitz et al., 1971; NIDA, 2017; Whitesell et al., 2012) and reservation living conditions, including intergenerational poverty, discrimination, and loss of culture (Brockie et al., 2013, 2015). Strength-based approaches that could delay onset or reduce problematic substance use are worthy of consideration given limited success of previous approaches over the past 30 years (Beauvais, 1998). Researchers have begun to respond to tribal communities call for strength-based cultural approaches for prevention and treatment (Gone & Calf Looking, 2011; Kana’iaupuni, 2005); however, the nature of culture as a protective factor against substance use has yet to be fully explored (Gone & Calf Looking, 2011).

Given the social and environmental conditions of reservation communities, and the within-group differences in substance use among Native American populations (IHS, 2012), Ungar’s definition and framework for resilience can be applied in this context (Ungar, 2011, 2012). Here, resilience is explored from an environmental perspective, whereby living conditions, including both social and political conditions that extend beyond the individual, extensively influence an individual’s resilience (Ungar, 2012).

In the context of exposure to significant adversity, resilience is both the capacity of individuals to navigate their way to the psychological, social, cultural, and physical resources that sustain their well- being, and their capacity individually and collectively to negotiate for these resources to be provided in culturally meaningful ways (Ungar, 2008).

The influence that specific aspects of culture and context have on health and illness in tribal communities needs to be further studied to best inform future prevention efforts.

Typically, culturally appropriate substance use programs for Native Americans consider community and culture (NIDA, 2012). However, there is a lack of available data evaluating these programs, and minimal rigorous studies exist which explore cultural factors that may be related to resilience — in a non-Western context and in non-Western populations. More specifically, identification of modifiable cultural protective factors for problematic substance use is critical for remote communities lacking access to specialty providers and quality care, including evidence-based, culturally appropriate substance use treatment programs. Interventions that are culturally appropriate are more well accepted and maybe more effective in addressing substance use among Native American youth (Beckstead et al., 2015; Donovan et al., 2015; Usera, 2017). As such, more effort is needed to adapt and evaluate evidence-based treatment and intervention programs.

Research has shown that specific aspects of traditional culture alone, or in addition to existing evidence-based practices, enhance substance use prevention among Native Americans, but the body of research shows mixed results. Traditional culture has been identified as a factor associated with decreased risk for substance use, and when incorporated into dialectical behavior therapy, overall improvement in externalizing and internalizing problems has been demonstrated (Beckstead et al., 2015). One study found Aboriginal identity and cultural participation served as a protective factor for 12-month prescription drug and illicit drug problems among Aboriginal adults in Canada, while acculturation served as a risk factor for prescription drug use (Currie et al., 2013). There are also mixed findings regarding the relationship between tribal identity and substance use among Native American adolescents (Donovan et al., 2015; Whitesell et al., 2014). Cwik and colleagues (2017) found high cultural identity was protective for binge drinking among White Mountain Apache adolescents. Meanwhile, others have found cultural identity to have no direct effect on substance use, specifically among adolescents attending off-reservation schools (Baldwin et al., 2011). Additionally, communal mastery has been found to be protective for smoking and drinking (Piko, 2006), depression symptoms, and anger in high-stress circumstances. Communal mastery, otherwise known as group efficacy, is a more appropriate resiliency measure for collectivist cultures. It has also been found to be more effective than self-mastery among samples of Native American women (Hobfoll et al., 2002).

There are few studies describing the prevalence and range of substance use in a high-risk reservation context and how cultural factors may influence use. Understanding how best to leverage and build upon existing cultural factors that bolster resilience and protect against substance use is a crucial step to developing effective, culturally appropriate intervention programs to reduce the burden of substance use among high-risk reservation communities. To address these gaps in knowledge, the purpose of this analysis was to: (1) describe the prevalence of substance use among a sample of Native American youth (15–24 years) on the Fort Peck Reservation in Montana, and (2) examine the protective role of cultural factors on polysubstance use in this high-risk, reservation-based setting. Specifically, we hypothesized that communal mastery, tribal identity, and education would be negatively associated with polysubstance use among this population.

## Methods

### Study Design and Sample

This study employed a cross-sectional correlational design. The research team employed a culturally grounded, community-based participatory research (CBPR) approach to build trusting, respectful relationships essential for conducting research on sensitive topics in Native American communities (Brockie et al., 2017). The full participation of Native researchers and the conscientious insistence on tribal engagement throughout the study provided a firm foundation for the long-term effort required to address salient issues. An all-Native American research team recruited a convenience sample of 288 participants in five of six communities from the Dakota (Sioux) and Nakoda (Assiniboine) Tribes from the Northern Plains Fort Peck Reservation in 2011. Once consented, participants entered data that was directly deposited into a web front hosted on a secure National Institutes of Health (NIH) server. The research team guided participants through enrollment, survey completion, and a debriefing. Participants were seated in every other workstation and alternating rows to ensure privacy. Cell phone use was not permitted. Participants logged on to a secure website to enter their survey responses from their individual workstation computer. As each participant finished, research staff verified survey completion before directing each participant to the debrief station. Research staff monitored the lab to help as needed and ensure each participant was fully logged out before exiting.

Tribal members 15–24 years of age, living within 50 miles of the local IHS Unit, were eligible to participate in the study. Verbal parental consent was obtained for participants 15–17 years of age, in person or via telephone. Participants completed an anonymous, web-based questionnaire on risk and protective factors for suicide and self-reported substance use. Participants received twenty dollars for survey completion. Tribal resolution authorized this study. Human subjects review was provided by the Johns Hopkins University (JHU) Institutional Review Board (IRB) and a reliance agreement, which is an authorization agreement for a single IRB review, was established between JHU and NIH. Fort Peck Tribal IRB provided review and approval for this manuscript.

### Measures of Interest

#### Substance Use

Frequency of use of inhalants, methamphetamine, marijuana, alcohol, and prescription drug misuse was assessed by questions adapted from the *National Youth Risk Behavior Survey* (YRBS) (Centers for Disease Control and Prevention [CDC], 2012). Questions included (1) “During your life how many days have you had at least at least one drink of alcohol?” with response options ranging from “I have never used alcohol”, to “1-2 times” to “100 times or more”; (2) “During the past 30 days, on how many days did you have a least one drink of alcohol?” with response options ranging from “0 days” to “all 30 days”. Prescription drug misuse was defined as any use other than for what it was originally intended.

Additionally, we asked which prescription drug was most misused, utilizing a list of drugs generated by community partners. Reliability of the YRBS has previously been established; 72% of the items are rated as having “substantial” or higher reliability (kappa = 61–100%) (Brener et al., 1995, 2013). Binge drinking was assessed by one question, “During the past 30 days, on how many days did you have five or more drinks of alcohol in a row, that is, within a couple of hours?” (Eaton et al., 2012). For each substance we asked, “How old were you when you first used [meth, inhalants, alcohol, marijuana, prescription drugs]?” Early initiation was defined as use of alcohol and illicit drugs at less than or equal to 14 years of age. Due to high prevalence of lifetime use of alcohol (87%) and marijuana (76%), the substance use variables were combined to create a polysubstance use variable, which was defined as: (1) those with lifetime alcohol use (> 100 times) and high lifetime marijuana use (> 100 times), and (2) at least one of three endorsements of lifetime inhalant, methamphetamine, or prescription drug misuse.

#### Childhood Trauma

The *Childhood Trauma Questionnaire* is a 28-item self-report inventory that provides brief, reliable, and valid screening for histories of abuse and neglect (Bernstein & Fink, 1998; Bernstein et al., 2003). The scale inquiries five types of maltreatment: emotional, physical, and sexual abuse, and emotional and physical neglect. Also included is a 3-item Minimization/Denial scale for detecting false-negative trauma reports. Internal consistency reports range from α=0.80 to 0.97, and its test-retest value for construct validity is 0.88.

#### Communal Mastery

Communal mastery was assessed utilizing the ten-item *Communal Mastery Scale* (CMS) (Hobfoll et al., 2002). Survey items included the following: “Working together with friends and family I can solve many of the problems I have,” and “I can do just about anything I set my mind to do because I have the support of those close to me.” Responses were ranked on a 4-point Likert-type scale (agree–disagree) (Jackson et al., 2000), and a total score was calculated by summing responses. Higher scores indicated higher communal mastery, despite reverse scoring for item 10. Cronbach’s alpha was .85 for this study.

#### Tribal Identity

Tribal identity was assessed using an adapted version of the 6-item *Oetting & Beauvais Orthogonal Cultural Identification Scale* (Oetting, 1997; Yoder et al., 2006), which measures extent of individual and familial involvement and perceived success in tribal culture. Our cultural adaptation specified “Assiniboine” and “Sioux” for tribal identity options, distinguishing tribal identity more specifically to make questions more relevant for the community. Survey items evaluated the extent participants’ families lived by Assiniboine or Sioux culture, how much the participants lived by Sioux or Assiniboine culture, how effective their families were in following Assiniboine or Sioux culture, and how effective participants would be, as adults, following Assiniboine or Sioux culture. Response options were ranked on a 4-point Likert-type scale (none–a lot). The scale score was computed by taking the mean for all six items, with higher values of the subscale indicating stronger identification with Assiniboine or Sioux culture. Cronbach’s alpha was .90.

### Data Analysis

Descriptive statistics were utilized to characterize the sample across several demographic variables including age, sex, school attendance, and level of education. Pearson’s chi-squared tests and *t*-tests were employed to determine the relationship between demographics and protective factors and frequency of substance use, polysubstance use, early initiation of substance use, and binge drinking. Additionally, a direct comparison of the prevalence of lifetime substance use among a subset of our sample (15–18 years) and that of the CDC YRBS from 2012 was evaluated using Pearson’s chi-squared tests.

A multiple logistic regression model was used to evaluate the relationship between protective factors (communal mastery, tribal identity, traditional activity, and spiritual activity) and polysubstance use, early initiation of substance use, and binge drinking, after controlling forage, school attendance (whether in school or not), and education (being at least a high school graduate). All protective factors with *p*<0.2 in preliminary bivariate analyses were entered into the model. A backwards elimination technique was used, removing probability of 0.1, to select the variables for the final model. All data analyses were performed using SPSS version 22. Significance was considered at the level of *p*<0.05.

## Results

### Sample Characteristics

Data was collected for 288 youth (female 52%; male 48%) participants. Characteristics of youth participants are outlined in Table 1. There were no notable differences in substance use by gender. The mean age of the sample was 19.25 years (female=19.23; male=19.27) and over half were 15–19 years of age (59%). Older participants (20–24 years) compared to younger individuals (15–19 years) were more likely to report methamphetamine use (*χ*^2^=27.3, *p*<.001), prescription drug misuse (*χ*^2^=7.9, *p*<.05), and to be categorized as a polysubstance user (*χ*^2^=9.6, *p*<.05). Participants not attending school, when compared to those attending school, had a higher percent of lifetime alcohol (*χ*^2^=4.2, *p*<.05), marijuana (*χ*^2^=7.1, *p*<.05), and methamphetamine use (*χ*^2^=8.2, *p*<.05), as well as prescription drug misuse (*χ*^2^=5.3, *p*<.05) and polysubstance use (*χ*^2^=13.4, *p*<.001). Nearly half of the sample (*n*=136, 47%) reported an annual family income of less than or equal to 2,500 dollars, a number consistent with the general assistance provided by the Bureau of Indian Affairs (2008). The unemployment rate was 70%, and overcrowding in housing, defined as greater than two people per bedroom, was 16% overall. Those living in overcrowded housing had a higher percent of inhalant use (*χ*^2^=4.3, *p*<.05).

**Table 1:** Characteristics of youth participants (*N* = 288)

	**Sample *n* (%)**
**Gender/sex**	
Male	139 (48)
Female	148 (52)
**Age group**^**D**, ^^e, f^	
15–19	169 (60)
20–24	115 (40)
**Tribal affiliation**	
Tribe 1	190 (66)
Tribe 2	96 (34)
**Residence**^**e**^	
West End	161 (57)
East End	123 (43)
**School attendance**^b, c, d^^, **F**^	
Student	137 (48)
Non-student	149 (52)
**Education**^d, e, f^	
< High school	200 (70)
≥ High School	84 (30)
**Employment**^d^	
Employed	63 (22)
Unemployed	115 (41)
Other	105 (37)
**Annual income**	
≤ $2500	136 (49)
> $2500	140 (51)
**Housing overcrowding**^a^	
≤ 2 ppb	238 (84)
> 2 ppb	44 (16)

*χ*^2^ significance at *p* < .05 (**caps/bold** if *p* < .001)

*ppb* people per bedroom

^a^inhalants

^b^alcohol

^c^marijuana

^d^methamphetamines

^e^prescription drugs

^f^poly-drugs

### Patterns of Substance Use

Again, the patterns of high-risk substance use are polysubstance use, early initiation of substance use, and binge drinking. Overall, youth most commonly reported lifetime use of alcohol (87%) and marijuana (76%). Meanwhile, 41% reported lifetime inhalant use, 33% reported prescription drug misuse, and 27% reported methamphetamine use (Table 2). Two-thirds of participants met criteria for polysubstance use (66%). Among those with *polysubstance use*, 118 (62%) reported inhalant use; 181 (95%) reported marijuana use; 75 (40%) reported methamphetamine use; and 93 (49%) reported lifetime prescription drug misuse, indicating use of substances across the spectrum. Approximately 35% of the sample reported illegal alcohol use (< 21 years of age) in the past 30 days. Second, the prevalence of *early initiation* of substance use (≤14 years) was as follows: inhalants (70%), alcohol (61%), marijuana (74%), methamphetamine (23%), and prescription drug misuse (23%). Finally, *binge drinking* was reported by 58% of the sample. Of those reporting prescription drug misuse, current use (within the past 30 days) was reported by 56% of individuals. Hydrocodone (opioid pain reliever) was endorsed as the most common misused prescription opioid (47%), followed by morphine (opioid pain reliever) (23%), suboxone (used to treat opioid addiction) (10%), and oxycodone (opioid pain reliever) (5%). Binge drinking was significantly related to polysubstance use (*χ*^2^=5.93, *p*<.05). Additionally, the relationship between early use of all substances and polysubstance use showed high levels of co-occurrence.

**Table 2:** Self-reported substance use (*N* = 288)

**Substance**	**Lifetime**	
	***n***	**%**
*Polysubstance use*	192	66.0
Inhalants	118	41.0
Alcohol	250	87.4
Marijuana	219	76.3
Methamphetamine	75	26.6
Prescription drug misuse	93	32.7

### Protective Factors

#### Communal Mastery

The communal mastery total score ranged from 12 to 40 (mean=30.9, SD=6.5). Females were more likely to score higher in communal mastery than males (*χ*^2^= 4.4, *p*<.05). Those with higher levels of communal mastery reported lower levels of polysubstance use (*β* =−0.057, *p*=0.013).

#### Tribal Identity

The tribal identity total score ranged from 0 to 3 (mean=1.67, SD=0.78). Of those who responded (*n*=273), 143 (50%) reported their family followed tribal culture “some” or “a lot” of the time. Approximately half of participants (52%) reported their family as being successful in tribal culture, and even more (65%) projected that in the future they would be successful in tribal culture. Higher levels of tribal identity were associated with lower polysubstance use *(β* = −0.584, *p*=0.0014).

### Multivariable Model

In the first multivariable logistic regression model, higher education, increased communal mastery, and high tribal identity were found to be significant protective factors against polysubstance use after controlling for age and school attendance (Table 3). Having at least a high school education was related to a 57% (OR=0.43, *p*=.028) lower odds of polysubstance use among participants after accounting for other variables in the model. After controlling for demographics and tribal identity, for every 1-point increase in communal mastery score, the odds ratio for polysubstance use decreased 7% (OR=.93, *p*=0.007). Similarly, after controlling for demographics and communal mastery, for every 1-point increase in tribal identity score, the odds ratio for polysubstance use decreased 42% (OR= 0.58, *p*<.009).

**Table 3:** Associations between demographic factors and polysubstance use

	**Model 1**							**Model 2**						
	B	S.E	Wald	*p*-value	OR	95% C.I. for OR		B	S.E	Wald	*p*-value	OR	95% C.I. for OR	
						Lower	Upper						Lower	Upper
Age	0.094	0.071	1.772	0.183	1.098	0.959	1.266	0.070	0.074	0.891	0.345	1.073	0.929	1.245
School attendance	0.586	0.391	2.247	0.134	1.797	0.835	3.901	0.504	0.418	1.456	0.228	1.655	0.729	3.780
Education	− 0.836	0.380	4.828	**0.028**	0.434	0.201	0.898	− 0.814	0.407	4.002	**0.045**	0.443	0.194	0.966
Communal mastery	− 0.071	0.026	7.318	**0.007**	0.931	0.882	0.979	− 0.061	0.028	4.649	**0.031**	0.941	0.887	0.992
Tribal identity	− 0.546	0.210	6.740	**0.009**	0.579	0.379	0.867	− 0.559	0.235	5.660	**0.017**	0.572	0.355	0.896
Trauma								0.028	0.013	4.779	**0.029**	1.029	1.005	1.058

In the second model, trauma was added as a predictor (Table 3). Education and tribal identity remained significant, and trauma was also found to be a significant predictor of polysubstance use. Having at least a high school education was related to a 56% (OR=.44, *p*=.045) decrease in the odds of polysubstance use among participants after accounting for other variables in the model. Everyone 1-unit increase in communal mastery was associated with a 6% (OR=.94, *p*=0.031) decrease in polysubstance use. For every 1-point increase in tribal identity score, the odds ratio for polysubstance use decreased 43% (OR=0.57, *p*=0.017). Lastly, increase in trauma noted a significantly higher odds for polysubstance use (OR=1.029, *p*<.029), after controlling for all other demographic and predictor variables. Finally, Table 4 shows the prevalence of various health risk behaviors in our sample compared with the CDC YRBS from 2012.

**Table 4:** Health-related risk behavior: Fort Peck (*N* = 288) compared to CDC YRBS†(*N* = 15,425)

	**Fort Peck**	**CDC**	
	**%**	**%**	***p*-value**
Ever inhalants	**41.6**	11.4	< .001
Ever alcohol	**82.3**	70.1	< .001
Drank alcohol for the first time before age 13 years*	**21.8**	20.5	= 0.205
Ever marijuana	**67.2**	39.9	< .001
Methamphetamines	**13.3**	3.8	= 0.002
Ever prescription drugs	**25.4**	20.7	= 0.224

^*^Fort Peck includes 15–18 years students and non-students: CDC YRBS 9th–12th grade students

^†^Centers for Disease Control and Prevention Youth Risk Behavior Survey (2012)

## Discussion

We explored patterns of polysubstance use in relation to communal mastery, tribal identity, and several demographics among young Native Americans living on the Fort Peck Reservation in the Northern Plains. We found tribal identity and having at least a high school education were associated with lower odds of polysubstance use, whereas early initiation was predictive of higher odds of polysubstance use. There is an urgent need to address substance use and misuse in this specific community as the reported lifetime use of methamphetamines (27%) was significantly higher than the overall national rate of 3.8% (CDC, 2012). Lifetime inhalant use was 2.5 to 4.5 times higher than that for Black students (9.2%), Hispanic students (14.4%) and White students (10.7%). Lifetime marijuana use (76%) was nearly twice that of White students (37.9%), for Black students (43.0%), and Hispanic (42.1%) students in grades 9 through 12. Lifetime alcohol use (87%) was higher than that of Black students (63.5%), Hispanic students (73.2%), and White students (71.1%) (CDC, 2012). The rate of prescription drug misuse (33%) was more than twice that of Black students (14.7%) and 10 percentage points higher than the rate for Hispanic students (19.4%) and White students (22.9%) in the same reporting year (CDC, 2012).

Substance use and age of initiation result from a complex interplay between youth, their environment, and the specific substance (Roberts et al., 2017; Whitesell et al., 2014). National studies support factors such as poverty, trauma history, poor school performance, peer drug use, and availability of drugs as risk factors for initiation of drug use (Stanley & Swaim, 2015). This has led to the development of programs like Project Venture and Living in 2 Worlds; however, existing programs primarily focus on those residing in urban areas (Carter et al., 2007; Kulis et al., 2017). To the authors’ knowledge, this analysis is one of the first to explore malleable and/or modifiable protective factors for Native youth living in a high-risk reservation environment. These study findings address a critical gap in understanding which could be leveraged to design or adapt existing programs for Native Americans living in different settings (Novins et al., 2011). A more proactive approach to addressing substance use in this population could have a drastic impact on preventing long-term challenges like sadness/hopelessness and youth suicide ideation/attempts which are directly linked to use of substances during adolescence (Manzo et al., 2015).

### Education

Staying in school and gaining at least a high school education was found to be protective for high-risk substance use in the bivariate analysis, and it remained significant in the final model. These findings corroborate similar findings for high-risk substance use among Native American adolescents (Tingey et al., 2016) and a national sample of high-risk communities (Sale et al., 2003). School is a logical setting to target youth for preventative, upstream efforts — before students dropout. Studies have identified involving youth as experts in adapting these types of programs can result in reduced substance use (Dunne et al., 2017).

### Communal Mastery

Promoting relationships and reconnecting youth with supportive family and community is a protective factor to leverage for addressing polysubstance use. We found significantly lower polysubstance use was associated with communal mastery at the bivariate level; however, this relationship did not remain significant once other factors were added. This may be related to shared variance with tribal identity or because of small sample size. Communal mastery, or group efficacy, is more aligned with tribal cultures than individually focused self-efficacy. It measures the extent to which youth gain strength and guidance through family and community connectedness. Communal mastery may lead to enhanced social connectedness and youth having mentors and role models that are able to address the challenges of adolescents and guide youth towards success in school and positively structured activities. Our findings are consistent with a study by Piko (2006), which found higher levels of communal mastery to be protective for adolescent boys and drinking behaviors.

### Tribal Identity

Dakota and Nakoda youth with stronger tribal identity had significantly reduced odds of polysubstance use at both the bivariate and multivariate levels. This is an important contribution to the literature, as enhancing and supporting tribal identity is a crucial factor for preventing high-risk substance use. Tribal identity measures the extent of individual and familial involvement in tribal culture and degree of success in following tribal culture. Contextually, tribal identity encompasses more than just identity; it includes a sense of belonging, shared values, traditions, spiritual practices, and language. Language gives meaning and understanding of tribal culture; it provides a pathway to spirituality, traditional knowledge, and worldview (Brockie et al., 2017). Our results are consistent with existing studies which found, among a sample of Native American college students, those who spoke their tribal language and felt traditional and spiritual values to be important. Additionally, those who participated in their tribe’s ceremonies and dances reported lower rates of past-month alcohol and drug use (Greenfield et al., 2018; Tingey et al., 2016).

There is a need for culturally appropriate alcohol and drug prevention and intervention programs for Native American youth (Dickerson et al., 2016). Through focus groups and interviews with Native American youth, parents, and providers, Dickerson and colleagues (2016) demonstrated the importance of integrating traditional activities with evidence-based treatments for alcohol and drug-related programs. As was suggested by Greenfield and colleagues (2018), a strong cultural base may also speak to social connectedness, connections to positive and supportive adults, and social support — all factors associated with lower likelihood of substance use disorders. Several studies have found that connectedness of an individual with their family, community, and environment is an important protective factor against substance use and suicide among Native American populations (Allen et al., 2006; Mohatt et al., 2004, 2011). According to Mohatt et al. (2011), culturally grounded protective factors are important because colonization disrupted connections to Native American traditions, and connectedness is related to hypothesized suicide and substance abuse protective factors, such as communal mastery, among Native American youth.

Notably, there was a bivariate relationship between traditional activity participation and polysubstance use using a categorical variable, although it did not hold significance in the final model. Future research is necessary to further explore these factors and develop valid measures. No relationship for spiritual activity participation and polysubstance use was found. This may be related to a lack of available, valid measures to holistically capture these constructs. Others conducting CBPR with Native American communities have identified this as a challenge, given that surveys and assessments are often developed outside of the target population of interest and may not be exactly aligned (Holliday et al., 2016). Nonetheless, we cannot underscore the authentic relationships developed through this process, which are pivotal to the success of any CBPR project, and ultimately contribute to the credibility of data produced (Brockie et al., 2017; Holliday et al., 2016).

## Limitations

The results of this analysis should be contextualized in terms of its limitations. First, the study was limited to a one reservation/two-tribe convenience sample, therefore, severely restricting generalizability to the broader U.S. Native American population. At the same time, it should be noted that this reservation is not unlike many isolated, rural reservations with similar socioeconomic profiles. Second, causal relationships cannot be inferred from the identified cross-sectional associations, particularly as data were self-reported and, thus, are subject to recall bias. However, the young age of this sample may minimize this concern. In addition, the way questions were posed in the CTQ presented a challenge in assessing the temporality of the constructs. Finally, no Bonferroni correction was used for multiple comparisons testing. Despite these methodological limitations, this study has several strengths. The study used a CBPR methodology, which was implemented by an all-Native American research team. Additionally, this is one of very few studies which examine associations between strength-based protective factors and high-risk substance use for Native American youth in high-risk reservation settings.

## Conclusion

The outlined findings speak to the importance of aspects of culture as key protective factors for mitigating high-risk substance use among at-risk Native American young people. In this sample, certain cultural factors such as communal mastery, tribal identity, and traditional activity participation were associated with less polysubstance use. Primary, secondary, and post-secondary educational institutions should thoughtfully consider how to support students in these cultural connections to foster positive health outcomes. Those developing and adapting substance use preventative intervention programs should intentionally integrate traditional practices and empirically test outcomes for Native American youth. To adequately investigate and address pathways to augment resilience in reservation-based youth, careful — and ongoing — consideration must be paid to the longstanding structural deficiencies among reservations and their visions of policies that are required to achieve health equity.
